# At-wavelength metrology of an X-ray mirror using a downstream wavefront modulator

**DOI:** 10.1107/S1600577524002157

**Published:** 2024-04-08

**Authors:** Tunhe Zhou, Lingfei Hu, Hongchang Wang

**Affiliations:** a Stockholm University Brain Imaging Centre, Svante Arrhenius väg 16A, Stockholm 11418, Sweden; b Diamond Light Source, Didcot, Oxfordshire OX11 0DE, United Kingdom; c National Synchrotron Radiation Laboratory, University of Science and Technology of China, Hefei, Anhui 230029, People’s Republic of China; IOM-CNR and Elettra-Sincrotrone, Italy

**Keywords:** X-ray mirror, speckle, metrology

## Abstract

At-wavelength metrology of X-ray optics plays a crucial role in evaluating the performance of optics under actual beamline operating conditions, enabling *in situ* diagnostics and optimization. Techniques utilizing a wavefront random modulator have gained increasing attention in recent years. However, accurately mapping the measured wavefront slope to a curved X-ray mirror surface when the modulator is downstream of the mirror has posed a challenge. To address this problem, an iterative method has been developed in this study. The results demonstrate a significant improvement compared with conventional approaches and agree with offline measurements obtained from optical metrology. We believe that the proposed method enhances the accuracy of at-wavelength metrology techniques, and empowers them to play a greater role in beamline operation and optics fabrication.

## Abstract 2

An iterative method to accurately map wavefront slope measurements from a downstream wavefront random modulator to a curved X-ray mirror surface is presented, addressing a significant challenge in at-wavelength metrology. The results show a substantial improvement over conventional approaches, enhancing the precision of at-wavelength metrology techniques for improved beamline operations.

## 1. Introduction

X-ray reflective optics are often used for focusing or collimating beams at synchrotron facilities, as well as laboratory X-ray systems. While pursuing diffraction-limited X-ray beams, the demand for the accuracy of polishing X-ray optics, as well as on metrology, is increasing. *Ex situ* metrology techniques (Nistea *et al.*, 2019 ▸), such as Fizeau interferometry, Diamond-NOM (Nanometre Optical Metrology), *etc*., are important for characterizing and preparing optics before installation. In order to investigate the X-ray optics’ behaviour under the actual working conditions, such as under high heat load (Rutishauser *et al.*, 2013 ▸) or being clamped (Xue *et al.*, 2019 ▸), *etc*., *in situ* and at-wavelength measurement is necessary. Moreover, with at-wavelength metrology, active tuning of bimorph mirrors (Sawhney, Alcock *et al.*, 2013 ▸) and optimization of alignment of optics (Zhou *et al.*, 2018 ▸) can be realized.

In recent years, speckle-based at-wavelength metrology using a wavefront random modulator (Sawhney, Wang *et al.*, 2013 ▸; Wang, Kashyap, Laundy & Sawhney, 2015 ▸; Kashyap *et al.*, 2016 ▸; Hu *et al.*, 2021 ▸) has attracted increasing attention due to its cost-efficiency, flexibility, fast acquisition and high resolution, compared with other existing methods, such as pencil beam (Hignette *et al.*, 1997 ▸), grating-interferometry (Bérujon *et al.*, 2012 ▸), ptychography (Kewish *et al.*, 2010 ▸) or Hartmann sensor (Idir *et al.*, 2010 ▸). The principle of speckle-based metrology is to use a wavefront modulator to generate speckle patterns, with which the deflection of the X-ray beam can be traced. Therefore, the wavefront, and hence the mirror surface slope, can be measured.

**Figure 1:**
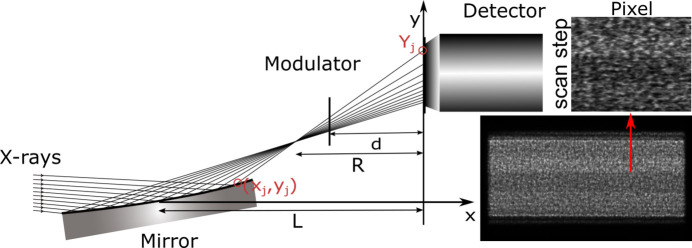
Illustration of the setup of the at-wavelength metrology technique using a downstream wavefront modulator.

One challenge for measuring focusing reflective optics with at-wavelength metrology is the non-linear relation between the coordinates on the mirror surface and the detector, as shown in Fig. 1 ▸ (Berujon & Ziegler, 2012 ▸; Zhou *et al.*, 2018 ▸). An iterative algorithm was developed for the technique using a wavefront modulator upstream of the tested optics (Berujon *et al.*, 2014 ▸). It has not yet been applied to a downstream modulator due to the different data processing procedures (Wang, Sutter *et al.*, 2015 ▸). Having the modulator downstream of the optics on the sample stage is not only practical and flexible but also sometimes the only solution after the beamline optics have already been installed. Additionally, it can provide higher sensitivity than the arrangement with the modulator upstream of the optics. Therefore, it is in demand to solve the non-linear mapping problem.

## 2. Methods

### 2.1. Theory

First of all, the principle of at-wavelength metrology using the scanning technique is briefly revisited (Wang, Kashyap & Sawhney, 2015 ▸). The setup is illustrated in Fig. 1 ▸. X-rays are reflected and focused by a mirror. Downstream of the mirror a wavefront modulator is scanned across the beam with a step size of μ. A series of images are recorded. The same row on the series of images forms a new figure. Two figures formed from adjacent rows are cross-correlated to evaluate the speckle pattern shift ɛ. The wavefront radius *R* of the X-ray beam can then be calculated using the geometric relation between the shift of the modulator ɛμ and the distance between the two rows (*m*, *n*) of pixels on the detector (Wang, Sutter *et al.*, 2015 ▸),

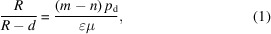

where *d* is the distance between the modulator and the detector and *p*_d_ is the pixel size. The wavefront slope φ can be calculated from (Wang, Sutter *et al.*, 2015 ▸)

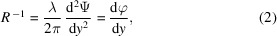

where λ is the wavelength and Ψ is the wavefront phase. We consider the one-dimensional focusing mirror here, therefore only *y* is considered.

Curved mirrors, such as spherical, parabolic or elliptical mirrors, do not reflect the incoming light in a linear fashion as flat mirrors. This is demonstrated by the simulated rays in Fig. 1 ▸. The outcoming light recorded on the detector, therefore, cannot be linearly projected back to the wavefront immediately before (or after) the mirror, nor to the mirror surface. To overcome this limitation, we modified the iterative algorithm from Berujon & Ziegler (2012 ▸) to the speckle-based technique where the modulator is downstream of the mirror. The local mirror slope *S* can be approximated as

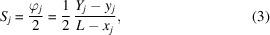

where *L* is the distance between the mirror centre and the detector, (*x*_*j*_, *y*_*j*_) is the coordinates of the mirror surface point *j*, and *Y*_*j*_ is the corresponding beam’s coordinate on the detector, as shown in Fig. 1 ▸. The mirror slope can be calculated from the wavefront slope φ_*j*_ and mapped to the mirror surface iteratively as

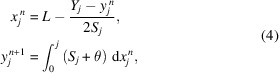

where θ is the incident angle.

Note that there are several points that distinguish our method from the upstream arrangement (Berujon & Ziegler, 2012 ▸). Although we use almost the same formulas, the parameters to be iterated are different. For the downstream scanning technique, we can determine the wavefront curvature definitively. The slope of the wavefront is calculated by integrating the measured local wavefront curvature. The mirror slope is then obtained using equation (3). As a result, the slope *S*_*j*_ is fixed, whereas in the upstream arrangement *S*_*j*_ is indefinite. On the contrary, in the downstream arrangement the local mirror coordinates (*x*_*j*_, *y*_*j*_) are both indefinite. This is in contrast to the upstream case, where the coordinate *y*_*j*_ is definite. However, similar to the upstream case, the number of iterative parameters in our method is also two.

Unlike the upstream arrangement (Berujon *et al.*, 2014 ▸), there is no direct coupling of coordinates between the incident beam and the reflected beam in the downstream arrangement, since all the measurements are conducted downstream of the mirror on the reflected beam, including the membrane stepping and image recording. If the tilt angle of the mirror or the distances of the membrane or detector were not accurately measured, the iterative procedure could converge to coordinates on a wrong part of the mirror. In order to avoid this, we firstly use a non-linear fitting as shown in equation (5) to find more accurate initial input for the iterative procedure by minimizing the wavefront radius difference between the measurement *R* and the simulation 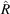 ,

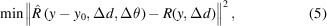

where *y*_0_ is the centre of the mirror, Δ*d* is the deviation of the distance between the modulator and the detector, and Δθ is the deviation of the mirror incline angle from the designed angle. For different mirror shapes the ray-tracing functions need to be adjusted. In this report we use an elliptical mirror as an example, for which the mirror is defined as follows,

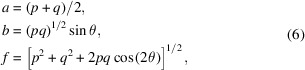

where *p*, *q* are the distances from the mirror centre to the source and the focus, respectively (Sutter *et al.*, 2010 ▸), *a* is the semi-major and *b* the semi-minor axis of the ellipse, and *f* is the focal length of the elliptical mirror.

**Figure 2:**
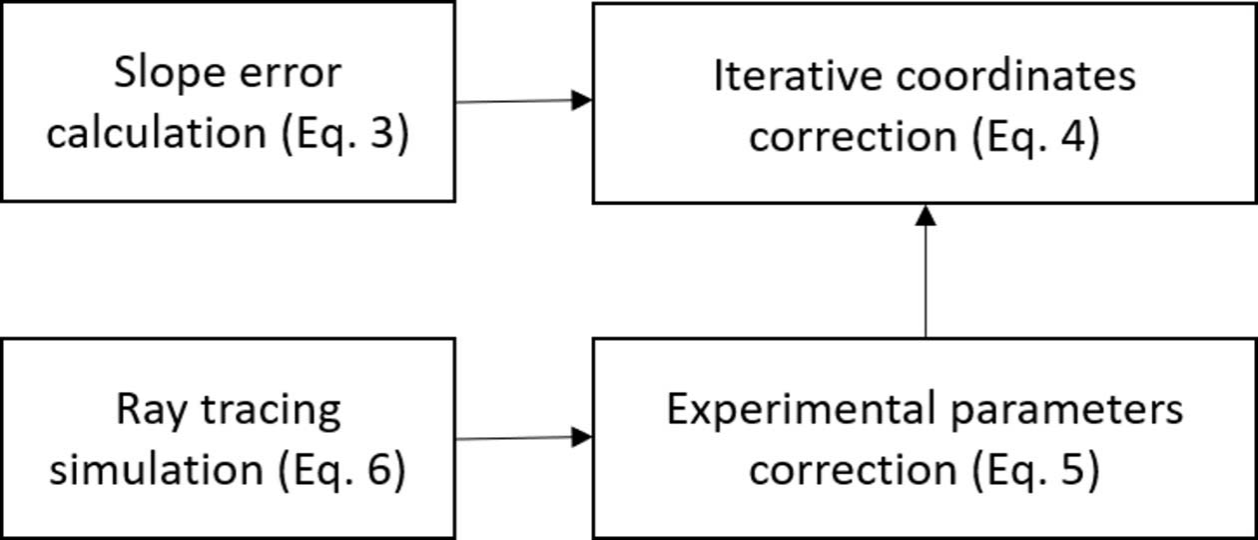
A brief scheme of the proposed method.

From the proposed method, the mirror centre, incident angle and the distance can firstly be corrected from measurement error using numerical simulation and fitting by equation (5), and the mirror slope will be updated iteratively to the correct coordinates on the mirror surface as in equation (4). Finally, the mirror slope error is the difference between the measured mirror slope and the theoretical mirror slope from simulation with designed parameters. The whole process can be summarized in the brief scheme shown in Fig. 2 ▸.

### 2.2. Experiments

Here we demonstrate the proposed method with experiments conducted at beamline B16 (Sawhney *et al.*, 2010 ▸) at Diamond Light Source. The mirror was a silicon elliptical mirror, with *p* = 46 m, *q* = 0.4 m, θ = 3 mrad. The mirror has additional parabolic arcs on top of the elliptical shape designed to purposefully change the size of the reflected X-ray beam (Laundy *et al.*, 2016 ▸). The energy of the X-rays was set to 9 keV by the double multilayer monochromator from a bending magnet source. The experimental setup is shown in Fig. 1 ▸ and consists of the mirror, P2000 sandpaper as the wavefront modulator, and a scintillator-coupled detector (Photonic Science FDS). The detector has a pixel size of 6.45 µm and was placed 5.1 m from the mirror. The mirror was mounted across the beam to reduce the influence of the horizontal striped wavefront errors from the upstream optics, such as the monochromator. For comparison, both upstream and downstream experiments were conducted with the same sandpaper and placed 0.4 m upstream or 1.1 m downstream of the mirror. During the experiment the sandpaper was stepped horizontally by a piezo stage with a step size of 0.06 µm for both upstream and downstream arrangement. In total, 181 images were acquired, each taking 0.1 s.

## 3. Results and discussion

**Figure 3:**
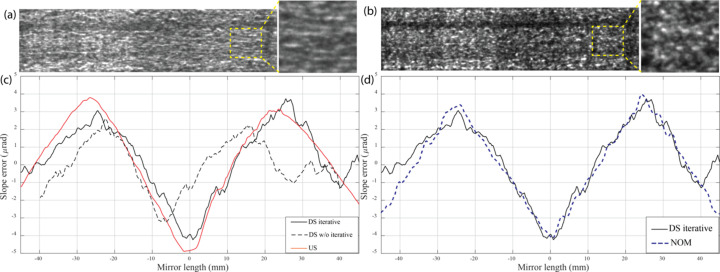
One image of the regions on the mirror that is used in the analysis from (*a*) with upstream (US) sandpaper, and (*b*) with downstream (DS) sandpaper. (*c*) Slope error from the experiments. The black line shows the measurement from the DS measurement with the proposed iterative methods, the red line from the US measurement, and the black dashed line from a previous linear approach of the mapping wavefront slope to the mirror surface without the iterative procedure. In panel (*d*) the slope error from Diamond-NOM is shown by the blue dashed line (Alcock *et al.*, 2010 ▸).

Images of the regions used in the analysis for both upstream and downstream arrangements are shown in Figs. 3 ▸(*a*) and 3(*b*). It can be seen that the speckle patterns from the downstream arrangement have a smaller size in the horizontal direction than for the upstream arrangement. This is because the speckles are magnified much more in the focusing direction in the upstream arrangement than in the downstream arrangement. The magnification factors are 13 and 4.6, respectively. A smaller speckle size is better for measurement sensitivity. For the upstream arrangement, this can only be achieved by moving the detector to reduce the propagation distance, but the visibility of the speckles is reduced as well, while for the downstream arrangement the magnification can be easily adjusted by moving both the detector and the sandpaper. Thus, the visibility and size of the speckles can be optimized.

A comparison of the speckle patterns with the modulator at different positions is shown in Fig. 3 ▸. The mirror-to-modulator distances of the three speckle images are 0.41, 0.54 and 1.19 m, respectively. It can be seen that the longer the mirror-to-modulator distance, the smaller the magnification factor in the horizontal direction. This can also be seen in Figs. 3 ▸(*d*)–3(*f*) through the normalized autocovariance calculated from equation (7), which is a popular method for quantifying the speckle sizes (Piederrière *et al.*, 2005 ▸),

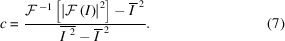

**Figure 4:**
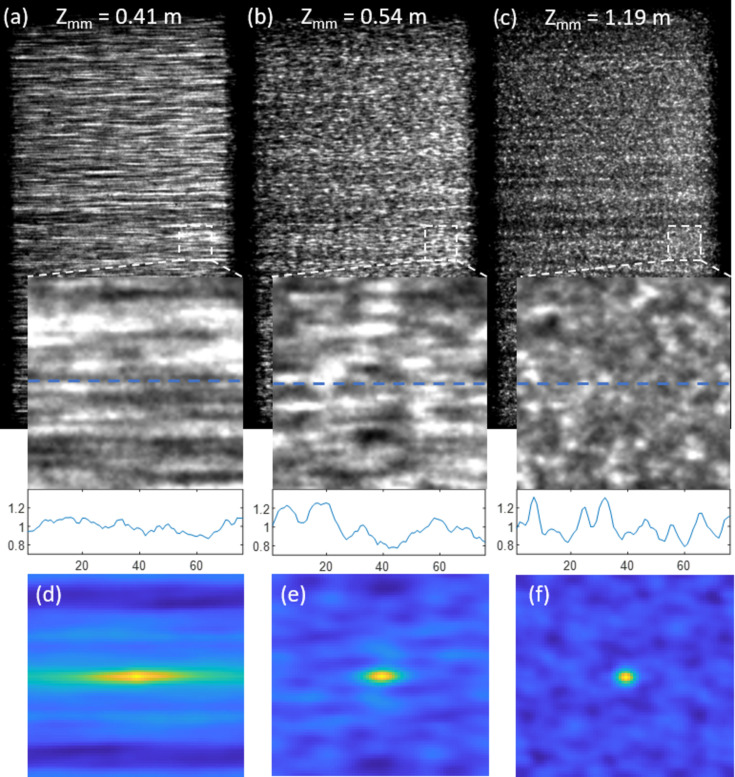
(*a*–*c*) Speckle images of an X-ray mirror at different mirror–modulator distances (*z*_mm_). Insets show the zoomed-in images of the white boxes. The line profiles show the normalized intensity from the position of the blue dashed lines in the insets. (*d*–*f*) Normalized autocovariance of the three insets showing the speckle sizes at the three distances.

The line profiles in Fig. 4 ▸ also indicate a smaller speckle size, as well as a better visibility of the speckles in the horizontal direction in panel (*c*) compared with (*a*) and (*b*). Therefore, it is not trivial to optimize the modulator position along the beam, which is only possible for the downstream rather than the upstream arrangement.

Slope errors from the experiments are shown in Fig. 3 ▸(*c*). The black line shows the slope error from the downstream measurement with the proposed iterative methods and the red line from the upstream measurement. It is obvious that the downstream measurement provides more sensitivity than the upstream one, where the high-frequency slope errors are smoothed out and only the two big peaks can be measured. The black dashed line shows the result of downstream measurement using the naïve linear projection back to the mirror surface without the proposed iterative algorithm. It is clear that without using the iterative method the linear approach could not map the result to the mirror surface correctly. In Fig. 3 ▸(*d*) the blue dashed line is the measured slope error from Diamond-NOM serving as a reference (Alcock *et al.*, 2010 ▸). Using the proposed iterative algorithm, the coordinates were mapped correctly and agree well with the results from NOM, except at the edges of the mirror, where the interference fringes at the mirror edges could not be separated from the mirror wavefront error using the at-wavelength methods. The root-mean-square (RMS) slope error of the iterative method is 1.88 µrad, while that of NOM is 2.32 µrad. Additionally, the peak-to-value (PV) slope error for each are 7.96 µrad and 8.70 µrad, respectively.

Discrepancies are also induced from the inhomogeneity of the mirror in the transverse direction, the influence of upstream optics in the beam, as well as the differences in resolution and sensitivity between the methods. The pixel size of the detector limits the spatial resolution of the retrieved sagittal wavefront slope, and the cross-correlation window width limits the resolution in the transversal direction. Considering the non-linear magnification of the beam for a focusing mirror, the mean effective pixel size is used here for simplicity to calculate the in-plane spatial resolution of the mirror as *p*_eff_ = 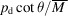 , with 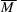 = 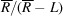 being the average magnification. For the downstream experiment reported here, *p*_eff_ was approximately 0.21 mm in the sagittal direction on the mirror surface. Using a cross-correlation window of 150 pixels in the transverse direction, the transversal resolution was therefore approximately the window width of 0.97 mm for both experiments. In comparison, the Diamond-NOM uses a 0.25 mm step size and a 3.5 mm-diameter autocollimator beam, which is the limitation of its in-plane spatial resolution (Alcock *et al.*, 2016 ▸). However, NOM has excellent sensitivity and stability with a sub-100 nrad repeatability. The sensitivity of the at-wavelength method was discussed by Wang, Sutter *et al.* (2015 ▸) to be

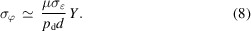

The sensitivity of detecting the modulator shift 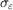 was found to be related to the speckle visibility, photon noise and correlation window size in a previous study (Zhou *et al.*, 2016 ▸). Here we estimate σ_ɛ_ as the standard deviation of the self-correlation of the speckle images: 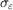 = 7.8 × 10^−4^ and the sensitivity of measuring the modulator shift to be μσ_ɛ_ ≃ 0.05 nm and σ_φ_ ≃ 12 nrad. This value is below the limit of the accuracy and stability of the linear stages; therefore, we can conclude that the stability of the *in situ* environment of the mirror and the experiment is the limiting factor for sensitivity of the at-wavelength metrology method and will be beamline-dependent.

## 4. Conclusion

In summary, we propose an iterative method to address the non-linear projection problem of at-wavelength metrology using a downstream wavefront modulator, aiming to achieve accurate mirror surface slope and figure error measurements. Experimental results obtained from an X-ray elliptical mirror demonstrate the effectiveness of our iterative method, showing a significant improvement compared with naïve linear projection. In contrast to the experimental arrangement with the wavefront modulator placed upstream of the mirror, the downstream arrangement offers higher sensitivity and greater flexibility by optimizing the setup. Consequently, we believe that the proposed at-wavelength metrology technique using a downstream wavefront modulator can serve as a valuable tool for *in situ* diagnosis, analysis and optimization of reflective optics.

Furthermore, the proposed iterative approach is not limited to wavefront modulators with random patterns but can also be applied to other techniques such as grating interferometry (Wang *et al.*, 2014 ▸). Similar to certain phase-contrast imaging methods where the second derivative and first derivative phase signals cannot be decoupled, resulting in fringes at the sample edges, our proposed method also exhibits such distortion at mirror edges. This limitation is inherent to *in situ* at-wavelength metrology compared with *ex situ* metrology.
